# Elevated de novo lipogenesis, slow liver triglyceride turnover, and clinical correlations in nonalcoholic steatohepatitis patients

**DOI:** 10.1016/j.jlr.2022.100250

**Published:** 2022-07-11

**Authors:** Eric J. Lawitz, Kelvin W. Li, Edna Nyangau, Tyler John Field, Jen-Chieh Chuang, Andrew Billin, Lulu Wang, Ya Wang, Ryan S. Huss, Chuhan Chung, G. Mani Subramanian, Robert P. Myers, Marc K. Hellerstein

**Affiliations:** 1 Texas Liver Institute, University of Texas Health Science Center San Antonio, San Antonio, Texas, USA; 2 Department of Nutritional Sciences & Toxicology, University of California Berkeley, Berkeley, California, USA; 3 Gilead Sciences, Inc., Foster City, Californi, USA

**Keywords:** Fatty acid synthesis, nonalcoholic fatty liver disease, NASH, tracer kinetics, stable isotope use in humans, triglycerides, de novo lipogenesis, mass spectrometry, acetyl-CoA carboxylase inhibition, ACC, acetyl-CoA carboxylase, ALT, alanine aminotransferase, APRI, AST to platelet ratio index, AST, aspartate transaminase, DNL, de novo lipogenesis, ELF, Enhanced Liver Fibrosis, GCMS, gas chromatography–mass spectroscopy, GGT, gamma-glutamyltransferase, IQR, interquartile range, MIDA, mass isotopomer distribution analysis, MRI-PDFF, magnetic resonance imaging–estimated proton density fat fraction, MRE, magnetic resonance elastography, NAFLD, nonalcoholic fatty liver disease, NASH, nonalcoholic steatohepatitis, TG, triacylglycerols

## Abstract

De novo lipogenesis (DNL) converts carbon substrates to lipids. Increased hepatic DNL could contribute to pathogenic liver triglyceride accumulation in nonalcoholic steatohepatitis (NASH) and therefore may be a potential target for pharmacological intervention. Here, we measured hepatic DNL using heavy water in 123 patients with NASH with fibrosis or cirrhosis, calculated the turnover of hepatic triglycerides to allow repeat labeling studies, and determined the associations of hepatic DNL with metabolic, fibrotic, and imaging markers. We found that hepatic DNL was higher in patients with fibrotic NASH [median (IQR), 40.7% contribution to palmitate (32.1, 47.5), n=103] than has been previously reported in healthy volunteers and remained elevated [median (IQR), 36.8% (31.0, 44.5), n=20] in patients with cirrhosis, despite lower liver fat content. We also showed that turnover of intrahepatic triglyceride pools was slow (t_½_ >10 days). Furthermore, DNL contribution was determined to be independent of liver stiffness by magnetic resonance imaging but was positively associated with the number of large very low density lipoprotein (VLDL) particles, the size of VLDL, the lipoprotein insulin resistance score, and levels of ApoB100, and trended toward negative associations with the fibrosis markers FIB-4, FibroSure, and APRI. Finally, we found treatment with the acetyl-CoA carboxylase inhibitor firsocostat reduced hepatic DNL at 4 and 12 weeks, using a correction model for residual label that accounts for hepatic triglyceride turnover. Taken together, these data support an important pathophysiological role for elevated hepatic DNL in NASH and demonstrate that response to pharmacological agents targeting DNL can be correlated with pretreatment DNL.

Hepatic de novo lipogenesis (DNL) is an essential biosynthetic pathway through which nonlipid energy substrates are converted to lipid species via the activities of several key enzymes, including acetyl-CoA carboxylase (ACC), ATP-citrate lyase, and fatty acid synthase (1, 2). Newly synthesized lipids generated by hepatic DNL are stored or excreted by hepatocytes and play key roles in hepatic energy homeostasis. The physiological roles and regulation of hepatic DNL in humans remain incompletely characterized, but elevated hepatic DNL may be important in a variety of metabolic disorders.

The most prevalent of these disorders is nonalcoholic fatty liver disease (NAFLD) (3, 4, 5). The global prevalence of NAFLD and its progressive form, nonalcoholic steatohepatitis (NASH), is estimated to be approximately 24% and 1.5%–6.5%, respectively, and is rising due to the epidemics of obesity and diabetes mellitus (6, 7). NASH is also emerging as a growing cause of hepatocellular carcinoma and will soon become the leading indication for liver transplantation (8). Hepatic steatosis, or accumulation of triacylglycerols (TGs), defines NAFLD and can in principle derive from three sources: fatty acids released through lipolysis of TG stored in adipose tissues, dietary TG carried in circulating lipoproteins, and hepatic DNL. Donnelly *et al.* reported that hepatic DNL in patients with NAFLD contributes up to 26% of liver TG compared with 2%–5% in normal patients and, thus, may be an important contributor to liver TG accumulation in this condition (9).

Progress in the development of pharmacologic inhibitors of key enzymes in the DNL pathway, such as ACC, positions hepatic DNL in humans as a target with practical importance for the treatment of NASH (4). Accurate direct measurement of hepatic DNL in vivo in humans for clinical trials is, however, challenging for several reasons. First, this is an intracellular biosynthetic process in the liver but needs to be assessed noninvasively; second, any in vivo metabolic labeling study requires knowledge of the label content in the true biosynthetic precursor pool in the tissue of interest (10); and third, the turnover of the intrahepatic TG storage pool is slow (11), which means that there may be label remaining in hepatic TG from a prior metabolic labeling study that could confound interpretation of a therapeutic intervention trial that involves a test/retest protocol.

A method for accurately measuring DNL using stable isotope tracers and mass spectrometry based on mass isotopomer distribution analysis (MIDA) and mathematical modeling was developed by our laboratory in the 1990s (12) and has recently been modified for longer-term labeling studies with heavy water (^2^H_2_O, deuterated water) (4, 9, 13). By this approach, very low density lipoprotein (VLDL)-TG secreted from the liver into the plasma is used as a window into the metabolic contribution from DNL to TG synthesized in the liver. The combinatorial isotopic labeling pattern in VLDL-TG-fatty acids, calculated by MIDA (12) after exposure to a stable isotope label such as ^13^C-acetate (incorporated via acetyl-CoA) or deuterated water (incorporated through the NADPH pool), allows accurate calculation of the fractional contribution from DNL to nonessential fatty acids. To account for potential delays in reaching plateau or near-plateau values for DNL due to the slow turnover of intrahepatic TG pools in NAFLD, heavy water is optimally administered as oral doses for days or weeks (4, 11). Heavy water labeling is safe, is relatively inexpensive, does not require an intravenous infusion, and can be readily administered for long periods on an outpatient basis, making it an attractive approach in this setting. Currently, there are no validated plasma lipid markers to replace direct measurement of hepatic DNL, although some studies have suggested that circulating fatty acid ratios might be useful as indirect markers of DNL (14, 15, 16, 17).

In the current study, we measured hepatic DNL using heavy water labeling in a large cohort of patients with NASH and fibrosis as baseline studies prior to participating in a 12 week clinical trial of pharmacological therapies. This approach addressed several important metabolic and study design questions that had not previously been resolved: the turnover time of the intrahepatic TG storage pool, based on the kinetics of label incorporation and die-away in plasma TG-palmitate; the contribution of hepatic DNL to circulating TG-palmitate at plateau after long-term metabolic labeling; and the optimal timing and calculation approach for repeat labeling studies in the setting of therapeutic interventions. We report that the hepatic TG storage pool in NASH turns over slowly; that the plateau DNL contribution to TG-palmitate is considerably higher than previously reported in studies that used shorter metabolic labeling periods; that DNL remains elevated in patients with cirrhosis despite reduced liver fat; and that use of a correction model for hepatic TG turnover permits repeat labeling studies after 4 weeks of an intervention. In addition, we explore associations of hepatic DNL with several biomarkers in patients with NASH.

## Materials and methods

### Patients and study design

We enrolled 123 patients with NASH-related fibrosis from 9 sites in the United States and 1 site in New Zealand in an open-label, proof-of-concept study including 9 treatment groups that evaluated the safety and efficacy of the ACC inhibitor firsocostat, the farnesoid X receptor (FXR) agonist cilofexor, and/or the apoptosis signal-regulating kinase 1 (ASK1) inhibitor selonsertib, given alone or in combination for 12 weeks (NCT02781584; full eligibility criteria and study design are detailed in the supplemental Material and supplemental Fig. S1). In the present analysis, we report baseline data from the 123 patients from all 9 cohorts as well as the posttreatment results for 10 patients with NASH-related cirrhosis and 10 patients with NASH-related fibrosis who were treated with firsocostat monotherapy. Some of the data (baseline and week 12) from the 10 noncirrhotic patients who received firsocostat monotherapy have been previously reported (4, 18). For the noncirrhotic cohorts (n=103), we enrolled patients 18–75 years of age with suspected NASH based on a clinical diagnosis of NAFLD plus a historical biopsy consistent with F2 to F3 fibrosis, according to the NASH Clinical Research Network classification (or equivalent), or a magnetic resonance imaging–estimated proton density fat fraction (MRI-PDFF) of ≥10% and liver stiffness by magnetic resonance elastography (MRE) of >2.88 kPa. Only patients without cirrhosis were eligible for enrollment in these cohorts, confirmed either by a FibroSure/FibroTest (LabCorp, Burlington, NC) result of <0.75 or a liver biopsy within 12 months of screening showing no evidence of cirrhosis.

For the cirrhosis cohorts (n=20), we enrolled patients with a clinical diagnosis of NAFLD plus a historical biopsy consistent with F4 fibrosis by NASH Clinical Research Network criteria (or equivalent), a historical liver stiffness by vibration-controlled transient elastography (FibroScan, Echosens, Paris, France) ≥14 kPa, or a liver stiffness by MRE ≥4.67 kPa during screening. Patients with body mass index (BMI) <18 kg/m^2^, serum alanine aminotransferase (ALT) concentration >5 times the upper limit of normal, serum creatinine concentration ≥2 mg/dl, or documented weight loss exceeding 5% between the date of the liver biopsy and screening were excluded (see supplemental Appendix for full eligibility criteria).

### Heavy water labeling protocol

**Fig. 1:**
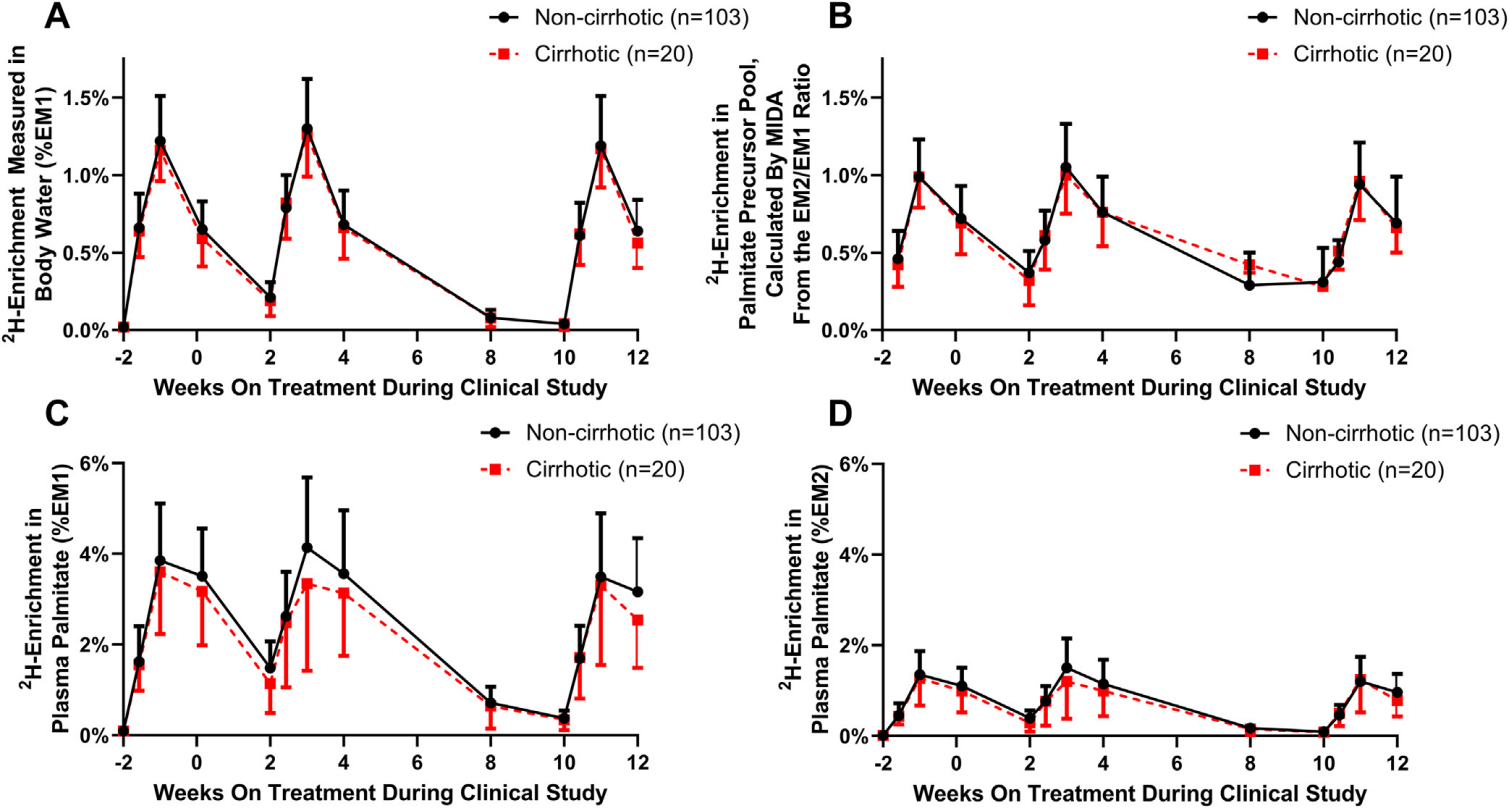
Isotopic enrichments in body water and plasma palmitate in NASH patient cohorts with and without cirrhosis. A: Oral administration of heavy water occurred over three labeling periods in the course of the study, resulting in ^2^H_2_O enrichment over natural abundance (EM1) in plasma. B: The isotopic enrichment of the palmitate precursor pool was determined by mass isotopomer analysis of the palmitate enrichment in the M1 and M2 isotopomers. C: ^2^H-enrichment of M1 isotopomer of palmitate in plasma samples collected over time was measured to calculate the palmitate precursor pool enrichment and hepatic DNL. D: ^2^H-enrichment of M2 isotopomer (%EM2) of palmitate in plasma samples collected over time was measured to calculate the palmitate precursor pool enrichment (datapoints are mean ± SD).

Patients underwent three cycles of heavy water administration: first during the 2 weeks prior to baseline and then prior to weeks 4 and 12 of treatment. Heavy water was administered orally in 1 week loading cycles (see Fig. 1A). The labeling protocol involved consumption of 50 ml of 70% deuterated water three times daily for 1 week, with blood samples collected 3, 7, and 14 days after the start of labeling. To confirm patient compliance, enrichment of deuterated water in blood samples was determined by gas chromatography–mass spectroscopy (GCMS) after reacting with acetone, as previously described (18). Body water ^2^H_2_O enrichment curves for the entire study are shown in Fig. 1A.

### Determination of hepatic DNL

The fractional contribution from hepatic DNL to palmitate (%DNL) in plasma was determined at the University of California, Berkeley in fasting blood samples collected during 14 day periods of heavy water exposure. To simplify sample processing, labeling of total plasma palmitate was used to represent hepatic DNL, based on a subset of fasting plasma samples collected in this study in which we observed that the isotopic enrichment of total plasma palmitate was quantitatively almost identical to that of palmitate isolated from VLDL-TG (details provided in supplemental Material). The time course of %DNL represents cumulative label incorporation across the circadian cycle in free-living patients over 2 weeks. Plasma palmitate was esterified and analyzed for mass isotopomer abundances by GCMS, using MIDA to determine the effective body water deuterium exposure (precursor pool enrichment) for the calculation of fractional DNL, as previously described (4, 10, 19). Briefly, equal parts chloroform and methanolic HCl were added to plasma samples and incubated for 1 h at 55°C to trans-esterify fatty acids to fatty acid methyl esters. Hexane, 3 ml, and 2 ml of water were added, mixed well, and centrifuged for 10 min at 2,500 RPM. The top organic layer containing the fatty acid methyl ester was transferred to another tube, to which was added another 3 ml hexane. The tube was centrifuged again, and the top organic layer was pooled with the organic phase of the previous extraction, dried under stream of nitrogen, and resolubilized in toluene for subsequent analysis by GCMS to quantify isotopic enrichment of both the M1 isotopomer and the M2 isotopomer due to the incorporation of deuterium from heavy water into palmitate. Specifically, a DB-17 (Restek RTX-50) or equivalent column was used, with electron impact ionization with single ion monitoring. Methyl-palmitate ions from palmitate-methyl esters were monitored at a mass-to-charge ratio of 270–272, representing the parent M0 through the M2 isotopomers. Excess M2 (EM2) and excess M1 (EM1) enrichments were determined by subtraction of natural abundance values in unlabeled standards (run in parallel) from the sample enrichment. The proportion of plasma palmitate that originated from the DNL pathway was then calculated from the EM1 and EM2 of palmitate using MIDA to determine both the biosynthetic precursor enrichment and the corresponding isotopic enrichment of newly synthesized palmitate molecules (10). The precursor pool enrichment (p) was determined from the ratio of EM2/EM1 in the experimental data. Knowledge of the calculated metabolic precursor pool enrichment and the known n (number of repeating subunits in the polymer = 21 for palmitate (20)) allows calculation of the theoretical asymptote enrichment of the single-labeled mass isotopomer species (EM1∗), representing the maximum possible enrichment when palmitate is newly synthesized at this deuterium precursor pool enrichment, as described previously (10). For samples collected during the baseline labeling period, the fractional synthesis of palmitate was then calculated by comparing the experimentally measured enrichment to the calculated asymptote:

(1) $$\text{Fractional Palmitate DNL}=\text{EM}1/{\text{EM}1}^{*}$$

Samples collected after an additional labeling period (W4 and W12 time points) required an adjustment to Equation 1 to account for labeled palmitate remaining from previous labeling periods (see below).

### Nonlinear regression analysis of the time course of DNL in NASH

The time courses of DNL during the baseline periods for each patient cohort, averaged across the 103 noncirrhotic patients and 20 cirrhotic patients, were analyzed by nonlinear regression (GraphPad Prism 8, GraphPad Software, San Diego, CA), using a two-phase exponential association model:

(2) $$\text{\%DNL}=\text{\%DN}{{\text{L}}_{\text{plateau}}}^{*}\left\{\text{\%}{\text{Fast}}^{*}\left[1-2^{\left(\frac{-\text{t}}{{\text{t}}_{1/2,\;\text{Fast}}}\right)}\right]-{\text{\%Slow}}^{*}\left[1-2^{\left(\frac{-\text{t}}{{\text{t}}_{1/2,\;\text{Slow}}}\right)}\right]\right\}$$

We used this two-phase exponential model to fit to the baseline study DNL datapoints and to characterize the relatively slow turnover pool that determines the half-life (t_1/2, Slow_, in days) by which %DNL approaches an equilibrium or plateau value (%DNL_plateau_) as well as the relative size of the fast (%Fast) and slow (%Slow) turnover pools. To constrain the two-pool model, a fast turnover pool with a fixed 3.3 h half-life (t_1/2, Fast_ = 0.14 days) was introduced to reflect the rapid turnover of TG in plasma (21).

### Measurement of the label die-away rate of the hepatic TG storage pool

**Fig. 2:**
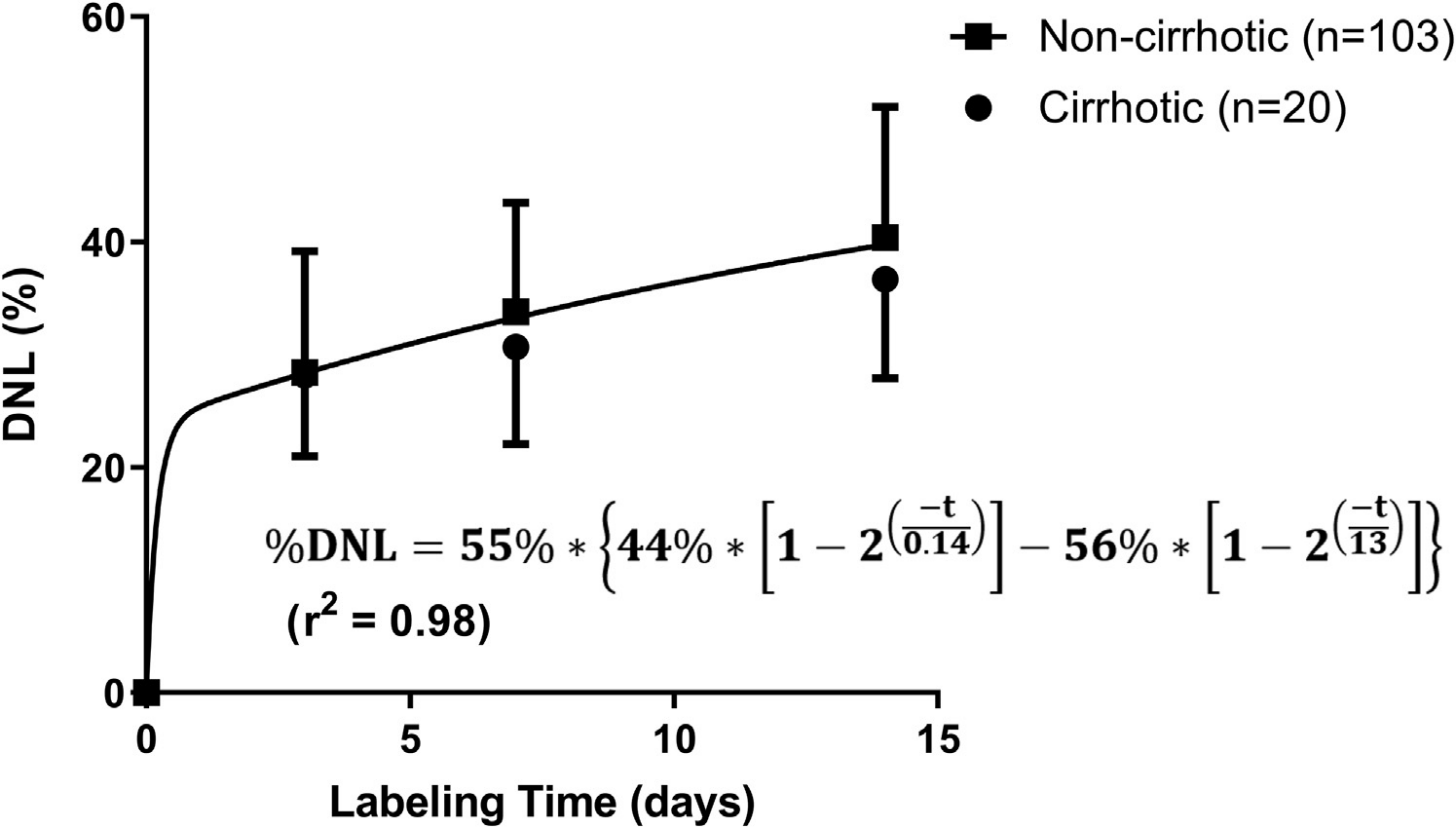
Baseline de novo lipogenesis (DNL) in NASH patient cohorts with and without cirrhosis rises over 14 days of isotopic labeling. A two-phase exponential curve fit (shown as the connecting line) was used to derive the rise-to-plateau kinetic parameters for the model equation shown (%DNL_plateau_ = 55%, %Fast = 44%, %Slow = 56%, t_1/2, slow_ = 13 days). Data are mean with error bars showing SD.

To quantitatively estimate in each subject the relatively slow hepatic TG turnover that was apparent in the label rise-to-plateau kinetics, we measured the die-away kinetics of labeled plasma palmitate after cessation of label exposure. Because the half-life of water in the human body is slow (Fig. 1A), we made measurements in blood samples between day 56 and day 70 of the study (week 8 and week 10, respectively, in Fig. 1), after body water deuterium enrichments had fallen to relatively low values. By this means, the die-away kinetics of the slow turnover pool of palmitate released from liver into blood can be assessed after a minimal correction for contamination by ongoing direct incorporation of new deuterium label from body water into palmitate in blood TG. In this model, the availability of heavy water tracer in body water at any given time contributes to plasma palmitate enrichment by a direct or immediate (fast turnover) pool and also much more slowly after passage through a slow turnover pool, as seen in the rise-to-plateau data here and as also previously shown by the delayed incorporation of ^13^C-acetate and labeled fatty acid tracers into blood TG in human subjects (11). Since the heavy water tracer present at the day 56 and 70 time points was small but not zero, it was necessary to impute the portion of the residual isotopic label found in plasma palmitate that was due to rapid palmitate labeling from the prevailing heavy water tracer (direct or immediate pool), such that the remainder can be attributed to having come from a slow turnover tissue storage pool. The two-phase exponential curve fit shown in Fig. 2 provided a determination of the relative contributions of the observed fast and slow pools to the isotopic labeling of plasma palmitate. Specifically, at both the week 8 and week 12 time points, the amount of isotopic enrichment attributed to the slow storage pool was determined by the following equation:

(3) $$\%EM1_{\text{slow, t}}=\%ΕΜ1_{\text{t}}-\left({ΕΜ1*}_{\text{BW}}\times \%{\text{DNL}}_{\text{W}12}\times \%\text{Fast}\right)$$

where

%EM1_t_ = the isotopic enrichment in plasma palmitate from plasma samples taken at time t (weeks 8 and 12 of treatment), which represents the summed combination of the isotopic label from newly synthesized palmitate and the residual isotopic label from labeling period at week 4.

%EM1_slow, t_ = the isotopic enrichment in plasma palmitate derived from a slow turnover tissue storage pool, at a given time point t.

EM1∗_BW_ = the asymptote enrichment of the single-labeled mass isotopomer species, representing the maximum possible enrichment when palmitate is newly synthesized, as a function of measured heavy water enrichment in the body water (BW) at time point t (10).

% DNL_W12_ = the fractional contribution of new hepatic DNL as determined from the corrected increase in isotopic enrichment (EM1) in plasma palmitate after the onset of repeat labeling with heavy water.

%Fast = the average relative size of the fast and slow pools, determined for the study cohort by two-phase exponential line fitting of the DNL time course at baseline (Fig. 2).

This calculation model assumes that fractional DNL contribution is stable from the week 8 and week 12 time points after an intervention. Using these values of %EM1_slow, t_, the half-life of labeled palmitate derived from the slow pool present at day 56 through day 70 was calculated using Equation 4, based on simple exponential decay:

(4) $${\text{t}}_{1\text{/}2}\;\left(\text{days}\right)\;=-14^{\text{∗}}\text{ln}\left(2\right)\text{ / ln}\left(\text{\%}{\text{EM}1}_{\text{slow, day }70}\text{ / \%}{\text{EM}1}_{\text{slow, day }56}\right),$$

where %EM1_slow, day 56_ and %EM1_slow, day 70_ represent the isotopic enrichments in plasma palmitate from the slow pool at study days 56 and 70, respectively.

### Individualized correction model for measuring hepatic DNL at weeks 4 and 12 of ACC inhibitor treatment

The turnover rate at which preexisting labeled palmitate in liver TG pools dies away has significant practical implications for the interpretation of new palmitate labeling during test-retest studies, when there is residual label in the precursor pool. This problem relates to body water enrichment in the present protocol, but the same principle applies for any repeated label study at the time of the repeat labeling study, which here was at 4 weeks or 12 weeks of treatment (Fig. 1A). If heavy water is readministered to patients when plasma palmitate still has residual isotopic enrichment from the previous labeling period, it is necessary to correct for the contribution from any residual palmitate enrichment present when calculating new DNL attributable to repeat heavy water exposure. It is intuitive that label present at the onset of a repeat heavy water administration (e.g., palmitate-EM1 at study week 2, Fig. 1C) should be measured, but a more difficult issue conceptually is how to correct for the enrichment from previously labeled palmitate that would have still been present at all of the various time points of the repeat labeling study. This value needs to be subtracted to accurately measure label that was incorporated only from the repeat protocol (Fig. 1C), but new label mixes with old label and is not physically distinguishable. Accordingly, a solution is to determine the rate at which the residual labeled palmitate dies away during the 2 week repeat labeling period, in order to calculate the proper amount of the total isotopic enrichment to subtract. For this purpose, we used the die-away rate of labeled palmitate in each individual patient, measured between days 56 and 70, in the calculation of new DNL during repeat labeling at weeks 4 and 12 of treatment. This individualized correction for residual label that was applied at the week 4 and week 12 labeling periods was implemented mathematically as shown in Equation 5:

(5) $${\%\text{DNL}}_{\text{W}4,12}=\frac{\text{\%EM}1_{\text{t}}-{\text{\%EM}1}_{\text{slow,}\text{t}0}*2^{\left(\frac{-\text{t}}{{\text{t}}_{1/2}}\right)}}{\text{\%EM}1^{\text{∗}}}$$

where

% DNL_W4,12_ = the fractional contribution of new hepatic DNL as determined from the corrected increase in isotopic enrichment (EM1) in plasma palmitate after the onset of repeat labeling with heavy water.

%EM1_slow, t0_ = the isotopic enrichment in plasma palmitate at the beginning of each repeat labeling period (study week 2 or 10) derived from the slow turnover tissue storage pool, determined in the patient using Equation 3.

%EM1_t_ = the isotopic enrichment in plasma palmitate from plasma samples taken at time t during the repeat labeling periods occurring at weeks 4 and 12 of treatment, which represents the summed combination of the isotopic label from newly synthesized palmitate during the repeat labeling period and the residual isotopic label from previous labeling periods.

%EM1∗ = the theoretical isotopic enrichment in newly synthesized palmitate under the measured integrated heavy water exposure (precursor enrichment), as derived using MIDA (10).

t = sample time relative to start of the repeat labeling period. Fasting plasma samples were drawn at days 3, 7, and 14 following the onset of the repeat labeling period.

t_1/2_ = half-life of palmitate in slow turnover pool measured from the exponential decay of previously labeled palmitate in the patient, according to Equation 4.

### Measurement of markers of hepatic steatosis, fibrosis, injury, and metabolism

Longitudinal changes in liver fat and liver stiffness from baseline to weeks 4 and 12 of treatment were assessed using MRI-PDFF and two-dimensional MRE (60 Hz), respectively. Assessments were performed by an experienced central reader blinded to clinical and histologic data. The methodology and assessments of changes in these parameters in colocalized regions of interest have been previously described (22, 23). Patients were categorized according to MRE-stiffness thresholds associated with fibrosis stage determined histologically (F0-F2: <3.64 kPa; F3: 3.64 to <4.67 kPa; and F4: ≥4.67 kPa) (24).

We also measured noninvasive markers of fibrosis at baseline, including the Enhanced Liver Fibrosis (ELF) test (Siemens, Tarrytown, NY), which includes tissue inhibitor of matrix metalloproteinase 1 (TIMP-1), procollagen III-N terminal peptide (PIII-NP), and hyaluronic acid (HA); FibroSure/FibroTest; the Fibrosis-4 index (FIB-4), and the aspartate transaminase (AST) to platelet ratio index (APRI) (25). In addition, we measured markers of liver injury and function [e.g., serum ALT), AST, bilirubin, gamma-glutamyltransferase (GGT), alkaline phosphatase, total bile acids, cytokeratin 18 M30 (CK18-M30), and CK18-M65]. Metabolic parameters measured at baseline included markers of glucose metabolism and insulin sensitivity [glucose, insulin, proinsulin, Homeostatic Model Assessment of Insulin Resistance (HOMA-IR), hemoglobin A1c (HbA1c)], cholesterol homeostasis [high density lipoprotein (HDL), low density lipoprotein, VLDL], apolipoproteins (apolipoprotein A1 and B), adipokines (leptin, adiponectin), and the Lipoprotein Insulin Resistance Score (LIPO-IR) (26), a composite metabolic marker of insulin-resistant dyslipoproteinemia.

### Statistical analysis

Associations between hepatic DNL with clinical parameters and other biomarkers at baseline were described using Spearman correlations, the corresponding *P*-values are based on approximate t-distribution. Between groups comparisons, including among patients with and without cirrhosis and according to MRE-stiffness categories, were made using the Wilcoxon rank-sum test (R version 3.6.0). The time courses of DNL during the baseline periods for each patient cohort were analyzed by nonlinear regression (GraphPad Prism 8, GraphPad Software, San Diego, CA), and the fit parameters were compared with each other by extra sum-of-squares F test. Repeated measures analysis of variance (ANOVA) was used to evaluate the longitudinal effects of treatment with the ACC inhibitor firsocostat on hepatic DNL, with post hoc Tukey testing (GraphPad Prism 8). Multiplicity adjustment was not performed based on the exploratory nature of this study.

### Study Oversight

The study protocol was approved by an Institutional Review Board/Independent Ethics Committee before study was initiated. The study was designed and conducted by the sponsor (Gilead Sciences) in collaboration with the principal investigators. Per study protocol, all investigators were responsible to ensure that the study was conducted in accordance with the principles of the Declaration of Helsinki (as amended in Edinburgh, Tokyo, Venice, Hong Kong, and South Africa), International Conference on Harmonisation guidelines, or with the laws and regulations of the country in which the research is conducted, whichever affords the greater protection to the study subject. These standards are consistent with the European Union Clinical Trials Directive 2001/20/EC and Good Clinical Practice Directive 2005/28/EC. The investigators ensured adherence to the basic principles of Good Clinical Practice, as outlined in 21 CFR 312, subpart D, “Responsibilities of Sponsors and Investigators,” 21 CFR, part 50, 1998, and 21 CFR, part 56, 1998.

## Results

### Patient characteristics

Baseline demographics and clinical characteristics of the 123 patients with NASH are summarized in Table 1. The median age was 56 years (interquartile range [IQR] 48, 63), median BMI was 35.5 kg/m^2^ (IQR 29.9, 41.4), 33% of patients were male, 42% were non-Hispanic, and 62% had diabetes mellitus. Compared with noncirrhotic patients (n=103), those with cirrhosis (n=20) had significantly higher liver stiffness by MRE, serum fibrosis markers including ELF (and its components), liver biochemistry (GGT, alkaline phosphatase, bilirubin), and serum bile acids, and lower platelets, large VLDL concentration, VLDL size, total HDL particles, and apolipoprotein A1 (Table 1).

**Table 1:** Baseline demographics and clinical characteristics of the study population

	All patients (n=123)	Patients without Cirrhosis (n=103)	Patients with Cirrhosis (n=20)	*P*-value^a^
Demographics				
Age, years	56 (48, 63)	55 (48, 61)	61.5 (55.5, 67)	0.01
Male	40 (33%)	33 (32%)	7 (35%)	N/A
Non-Hispanic ethnicity	52 (42%)	41 (40%)	11 (55%)	N/A
Diabetes	76 (62%)	66 (64%)	10 (50%)	N/A
BMI, kg/m^2^	35.48 (29.88, 41.35)	36.25 (31.2, 41.83)	30.39 (28.2, 37.13)	0.02
Liver Biochemistry				
ALT, U/L	50 (35, 81)	50 (36, 81)	44 (32, 68)	0.39
AST, U/L	44 (31, 65)	42 (30, 68)	49 (43, 61)	0.14
GGT, U/L	57 (36, 96)	55 (34, 86)	96.5 (55.5, 163)	0.00
Alkaline phosphatase, U/L	86 (71, 114)	84 (70, 106)	111.5 (76.5, 128.5)	0.04
Albumin, g/dl	4.4 (4.3, 4.7)	4.4 (4.3, 4.7)	4.5 (4.1, 4.7)	0.52
Platelets, x10^3^/μL	207 (167, 258)	219 (176, 274)	167 (142.5, 182)	<0.001
Bilirubin, mg/dl	0.5 (0.3, 0.7)	0.4 (0.3, 0.6)	0.65 (0.5, 1)	<0.001
Total bile acids, μmol/L	6.35 (4.9, 11.4)	6.15 (4.9, 9.25)	11.55 (6.25, 29.05)	<0.001
Markers of Hepatic Steatosis, Fibrosis, and Injury				
MRI-PDFF, %	15.9 (11.7, 20.1)	16.5 (13.3, 20.5)	9.1 (5.2, 13.2)	<0.001
Liver stiffness by MRE, kPa	3.74 (3.15, 4.74)	3.56 (3.12, 4.21)	6.71 (5.47, 7.35)	<0.001
FIB-4	1.57 (1.07, 2.54)	1.42 (1.02, 2.18)	2.58 (2.05, 3.64)	<0.001
FibroSure/FibroTest	0.3 (0.16, 0.5)	0.27 (0.15, 0.43)	0.66 (0.4, 0.78)	<0.001
APRI	0.63 (0.38, 1.04)	0.55 (0.34, 0.96)	0.99 (0.69, 1.29)	0.002
ELF	9.78 (9.12, 10.63)	9.65 (9.06, 10.37)	11.07 (10.18, 11.89)	<0.001
Hyaluronic acid, ng/ml	66.31 (34.85, 125.01)	56.67 (34.05, 95.98)	156.95 (76.92, 307.67)	<0.001
PIII-NP, ng/ml	10.61 (8.28, 14.04)	10.12 (7.9, 13.34)	16.31 (10.77, 25.98)	<0.001
TIMP-1, ng/ml	277.2 (232.2, 334.7)	265.8 (225.9, 311.6)	350.7 (297.7, 402.75)	<0.001
CK18M30, U/L	346 (192, 662)	349 (189, 645)	314 (226.5, 795)	0.67
CK18M65, U/L	509 (234, 1159)	527 (197, 1159)	483 (304, 1317)	0.49
Metabolic Markers				
Glucose, mg/dl	114 (97, 149)	116 (99, 149)	104.5 (95.5, 145.5)	0.37
HOMA-IR	6.81 (4.3, 10.31)	6.81 (4.3, 10.21)	7.55 (4.29, 13.39)	0.50
HbA1c, %	6.2 (5.6, 7.3)	6.3 (5.7, 7.3)	5.95 (5.3, 7.05)	0.39
Insulin, μIU/ml	21.83 (15.32, 33.6)	21.76 (15.13, 33.08)	26.89 (16.45, 40.17)	0.30
Proinsulin, pmol/L	14.6 (8.5, 29)	14.2 (8.2, 29)	14.9 (10.1, 30.05)	0.66
TG, mg/dl	156 (119, 226)	156 (120, 224)	147 (104, 240.5)	0.31
HDL-C, mg/dl	46 (37, 54)	46 (37, 54)	43.5 (35.5, 52.5)	0.57
Non-HDL cholesterol, mg/dl	129 (110, 152)	130 (111, 152)	115.5 (98.5, 157)	0.42
VLDL-TG, mg/dl	86 (56, 134)	86 (61, 134)	81.5 (40, 128)	0.26
Total HDL particles, μmol/L	29.2 (24.5, 33.2)	29.8 (24.9, 34.4)	25.35 (22.85, 29)	0.00
Total LDL particles, nmol/L	1302 (1021, 1530)	1308 (1068, 1518)	1159.5 (931.5, 1557)	0.38
Total VLDL & chylomicron particles, nmol/L	44.6 (28.8, 72.5)	44.6 (29.8, 68.6)	45.15 (22.7, 88.6)	0.99
ApoA1, mg/dl	136 (125, 159)	140 (125, 162)	131 (120, 138)	0.05
ApoB, mg/dl	93 (77, 108)	94 (80, 107)	84.5 (74.5, 113.5)	0.60
LIPO-IR	64 (52, 74)	65 (56, 75)	56 (39, 66)	0.01
Large VLDL, nmol/L	5.3 (3.6, 9.1)	5.5 (3.8, 9.2)	3.75 (1.8, 6.45)	0.02
Medium VLDL, nmol/L	16.6 (6.1, 35)	16.9 (8.5, 32.5)	14.3 (4.85, 39.7)	0.88
Small VLDL, nmol/L	19.7 (11.1, 33.1)	18.9 (9.8, 32.4)	21.25 (14.4, 41)	0.19
VLDL size, nm	54 (49.6, 58.3)	54.3 (50.6, 59.1)	49.45 (45.7, 54.8)	0.00
Adiponectin, ng/ml	3555.55 (2370.7, 5046.3)	3649.7 (2370.7, 5181.4)	2907.65 (2292.25, 4643.65)	0.39
Leptin, pg/ml	31,726.7 (15,720.58, 45,958.87)	32,440.75 (16,636.19, 46,934.41)	28,029.86 (12767.72, 39829.4)	0.32
Free fatty acid, mEq/L	0.6 (0.4, 0.8)	0.6 (0.4, 0.7)	0.6 (0.3, 0.85)	0.90
Beta-hydroxybutyrate, mg/dl	0.9 (0.9, 1.2)	0.9 (0.9, 1.1)	1.1 (0.9, 1.45)	0.03

Data are median (Q1, Q3) or n (%).

a *P*-values for comparison of patients with and without cirrhosis.

### Hepatic DNL in patients with NASH

#### Time course of metabolic labeling of plasma palmitate

Among 123 patients with NASH, the administration of three courses of heavy water as a metabolic tracer (Fig. 1A) resulted in a time course of labeling of newly synthesized palmitate found in the plasma, as quantified by isotopic enrichment in the M1 and M2 isotopomers of palmitate (Fig. 1C, D). The measured EM1 and EM2 enrichments in palmitate were used to determine the effective precursor pool enrichment during biosynthesis over the labeling periods (Fig. 1B) by MIDA. The MIDA-calculated precursor pool value represents the integrated average of the precursor enrichment available at the biosynthetic dates for the palmitate molecules that are present in a sample and are therefore in principle more appropriate than body water enrichments (Fig. 1A) for calculating fractional replacement in this study (10).

During the baseline period (from study day -14 to 0), cumulative hepatic DNL over 14 days was elevated above that previously observed in healthy controls measured by similar heavy water labeling protocols (4, 27) but was not different between the patients with NASH with and without cirrhosis (Fig. 2). Notably, the contribution of hepatic DNL to plasma palmitate had not reached a plateau at 14 days after the onset of heavy water labeling. Quantitatively, the two-phase exponential fits in the noncirrhotic and cirrhotic cohorts were not markedly different from each other (*P*=0.17), and the shared best-fit parameters indicated that the %DNL would reach a steady-state plateau of 55%, with a fast phase pool that provided 44% of the DNL contribution to plasma palmitate, and a slow phase pool that turned over with a half-life of 13 days and provided 56% of the DNL contribution to plasma palmitate. These observations are consistent with a general model that in NASH subjects there is a fast-turnover hepatic TG pool with a half-life of hours and a much slower turning over TG pool with a half-life in excess of 10 days.

#### Measurement of the label die-away rate of the hepatic TG storage pool

**Fig. 3:**
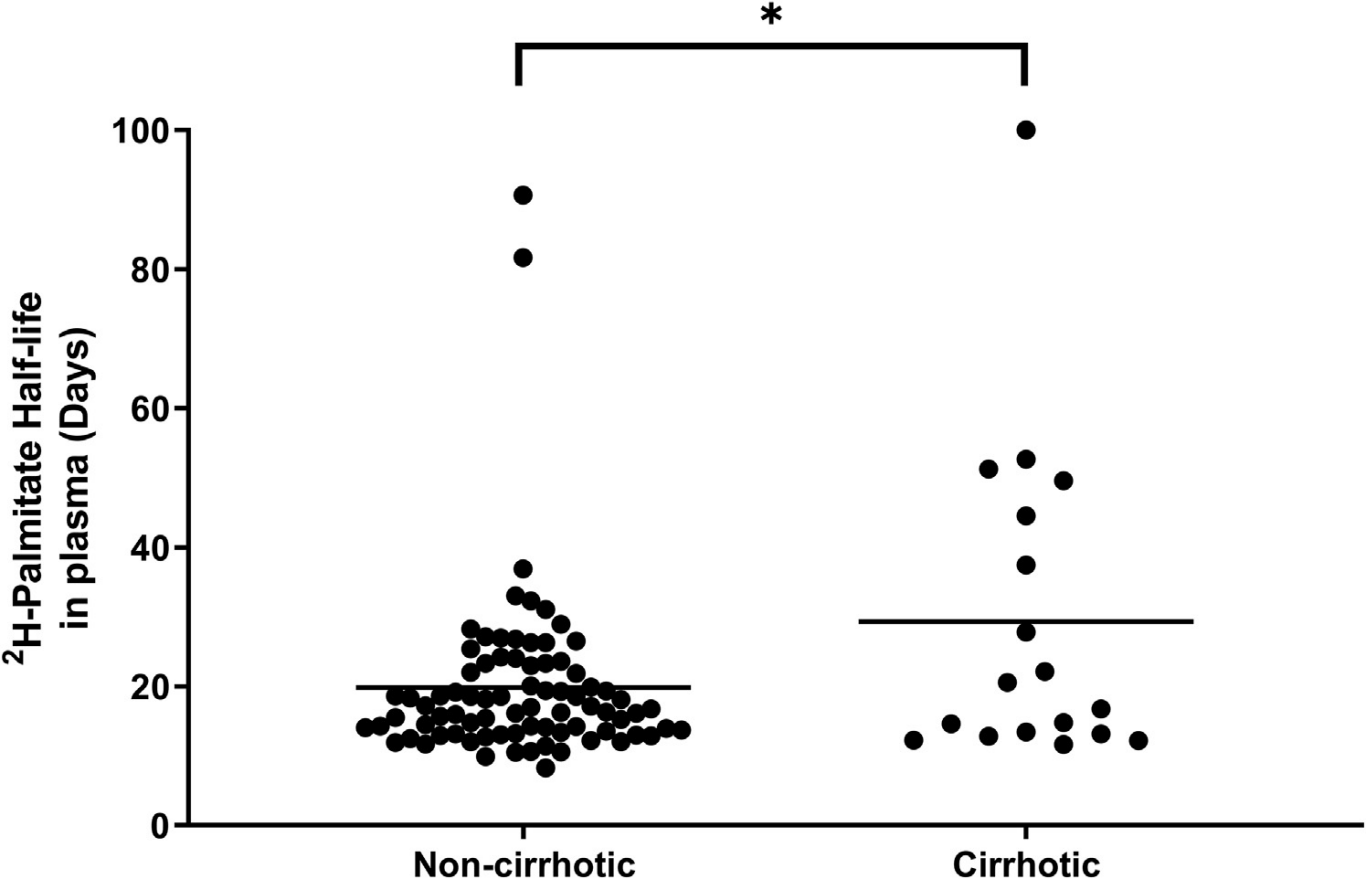
Direct measurement of the turnover of labeled palmitate in patients with NASH with and without cirrhosis. Palmitate enrichment measured from day 56 to day 70 of the study in the near-absence of heavy water enrichment (see Fig. 1A) that includes a correction for low ongoing label incorporation, indicates a hepatic storage pool of TGs that turns over slowly, and varies between individual patients. Calculated turnover rate of hepatic TG is significantly different between cirrhotic (n=18) and noncirrhotic (n=103) patients with NASH (∗ *P* =0.016).

To further elucidate the hepatic TG turnover rate estimated from the label rise-to-plateau kinetics in the group, we also measured the individual die-away kinetics of isotopic enrichment in plasma palmitate in each subject after cessation of label exposure. Specifically, based on measurements from samples taken between days 56 and 70 of the study, we directly assessed the die-away kinetics of the slow turnover pool of liver palmitate after body water deuterium enrichments had fallen to low levels (Fig. 1A). This analysis indicates that in patients with NASH with and without cirrhosis, there is a pool of palmitate that turns over with a half-life averaging 17 and 25 days, respectively (*P*<0.01 for comparison between noncirrhotic and cirrhotic patients with NASH; Fig. 3). This slow-turnover pool half-life exhibited significant variability among patients (IQR of 12–21 days in NASH; n=80), which overlaps with the 13 day half-life derived from two-phase exponential modeling of the rise-to-plateau %DNL time course during baseline labeling (Fig. 2). We have shown elsewhere (27) that DNL from adipose-derived fatty acids contributes minimally to DNL that is measured in plasma palmitate even during a long-term labeling period of 3–5 weeks duration, so the labeled slow turnover pool is not derived from adipose tissue, but must derive from TG in the liver.

#### Use of the individualized correction model for effects on hepatic DNL at Week 4 of treatment

**Fig. 4:**
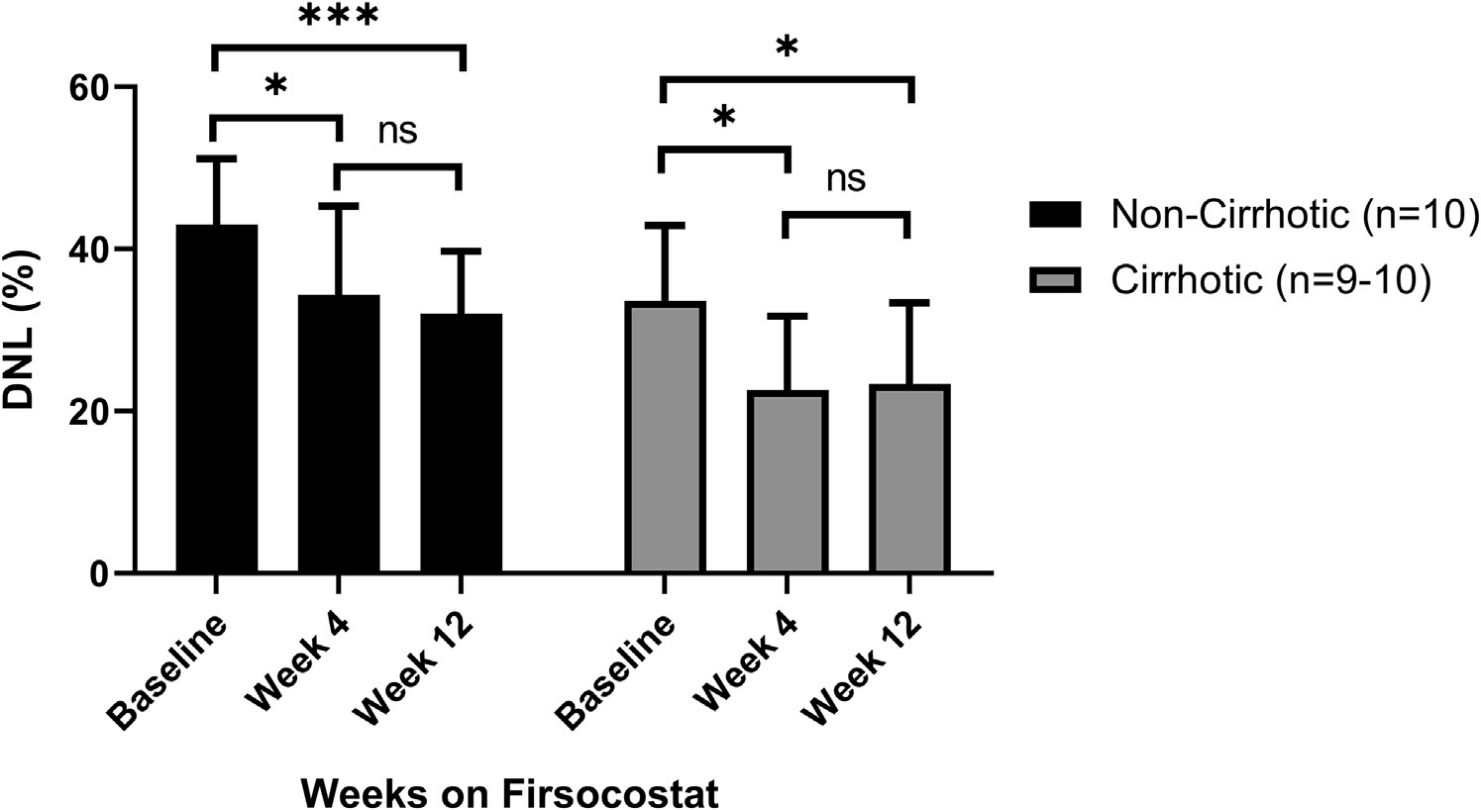
De novo lipogenesis (DNL) in patients with NASH with or without cirrhosis, given firsocostat for 12 weeks, with repeat measurements of DNL at weeks 4 and 12 of treatment. ∗*P*<0.05, ∗∗∗*P*=0.003. Data are mean + SD. Week 12 data from the noncirrhotic cohort have been previously published (4).

The measurement of a slow turnover rate for a portion of the newly synthesized palmitate enabled a correction model to be used for repeat labeling experiments that accounted for the impact of residual palmitate label in each individual subject. Use of the individualized correction model for treatment week 4 measurements of DNL gave very similar results to those from DNL measurements at treatment week 12 (*P*=0.7 and 0.9 in the noncirrhotic and cirrhotic patient cohorts, respectively; Fig. 4), even though the week 12 measurements were negligibly contaminated by residual label, whereas the treatment week 4 measurements still had substantial contributions from previously labeled palmitate present. These findings demonstrate that treatment effects can be assessed as early as 4 weeks after initiation of therapy by correcting for residual label content and die-away rate. Moreover, treatment with the ACC inhibitor firsocostat in patients with NASH with either cirrhosis or fibrosis was effective in reducing hepatic DNL at both week 4 (*P*=0.02; Fig. 4), using this individualized correction factor, and at week 12 of treatment (*P*=0.04).

#### Associations between hepatic DNL and fibrosis markers in NASH

**Fig. 5:**
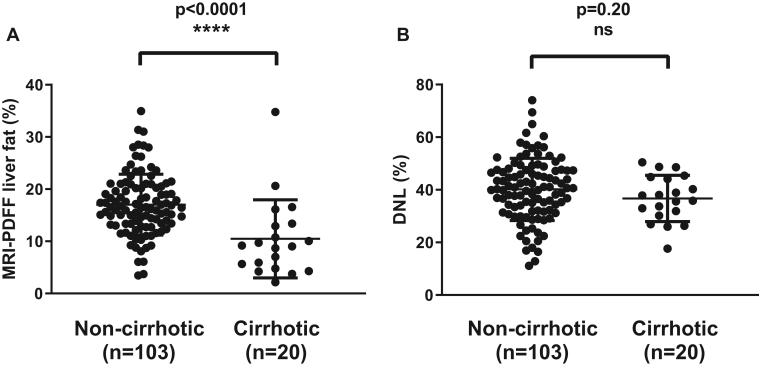
Baseline liver fat and de novo lipogenesis (DNL) in patients with NASH with and without cirrhosis. A: Patients with NASH with cirrhosis have lower liver fat content by MRI-PDFF compared with patients without cirrhosis. B: Hepatic DNL is not significantly different in patients with NASH with and without cirrhosis.

At baseline, patients with NASH with cirrhosis had significantly lower median (Q1, Q3) hepatic fat content as measured by MRI-PDFF than those without cirrhosis (9.1% [5.2, 13.2] vs. 16.5% [13.3, 20.5]; *P*<0.0001) (Fig. 5A). However, hepatic DNL did not differ between groups (36.8% [31.0, 44.5] vs. 40.7% [32.1, 47.5]; *P*=0.20) (Fig. 5B). Hepatic DNL also was not correlated with MRE-stiffness at baseline (r=-0.07; *P*=0.49) and was similar between groups categorized according to MRE-stiffness thresholds (<3.64 kPa vs. 3.64 to <4.67 kPa vs. ≥4.67 kPa: 39.9% vs. 43.6% vs. 35.6% [*P*=0.09]). Although hepatic DNL and ELF score were not correlated in noncirrhotic patients (r=0; *P*=0.98), a negative correlation was observed in those with cirrhosis (r=-0.52; *P*=0.019). Similar trends to negative correlations with DNL were observed for ELF components (HA, PIIINP, and TIMP-1), FIB-4, and platelets (supplemental Table S1).

#### Associations between hepatic DNL and other clinical parameters in NASH

Hepatic DNL was not significantly associated with demographic factors (e.g., age, gender, body weight, BMI), liver biochemistry, or hepatic fat as estimated by MRI-PDFF (r=0.03; *P*=0.78) (supplemental Table S1). Hepatic DNL was positively correlated with multiple lipid parameters including circulating TG (r=0.28; *P*=0.0014), total VLDL concentration (r=0.29; *P*=0.0014), number of large VLDL particles (r=0.42; *P*<0.0001), and VLDL size (r=0.41; *P*<0.0001) (supplemental Table S1). While hepatic DNL was not correlated with fasting free fatty acids (r=-0.14; *P*=0.12), significant associations were observed with beta-hydroxybutyrate (inverse, r=-0.27; *P*=0.0023) and the LIPO-IR score. HOMA-IR (r=0.18; *P*=0.05), fasting glucose (r=0.16; *P*=0.085), and proinsulin (r=0.20; *P*=0.024), but not HbA1c (r=0.04; *P*=0.69), were weakly associated with hepatic DNL.

## Discussion

This study addressed several questions related to the role of hepatic DNL in NASH, its measurement, and the impact of treatment.

### Slow time course of label rise-to-plateau and label die-away in hepatic TG pool in NASH and high plateau values of DNL contribution

The finding of slow turnover kinetics from both labeling and delabeling of plasma palmitate strongly suggests that the TG pool in the liver of patients with NASH turns over slowly. The presence of a slow-turnover hepatic TG pool in NASH means that short-term metabolic labeling protocols to measure DNL will in principle underestimate the DNL contribution due to the continued presence of unlabeled TG-palmitate that was synthesized before the onset of label exposure. Consistent with this kinetic finding, we report hepatic DNL fractional contribution values in patients with NASH of ∼40%, which are higher than previous reports in NAFLD of 15%–26% DNL contribution based on labeling periods of 1–4 days duration (9, 20). Lambert *et al.* (3) reported DNL values of 23% in NAFLD from 9 days of heavy water labeling, but they subtracted ∼6% for possible DNL contribution from adipose tissue, which we have since shown to be negligible (27), and their measurement of DNL was after a longer overnight fast, which they showed progressively reduced DNL contribution.

It is worth noting that MIDA-calculated precursor pool values (p) represent the integrated average, or “effective,” precursor enrichment present at the biosynthetic sites and dates for the palmitate molecules that are present in a sample. Inference of effective precursor pool enrichment is important in the context of a body water enrichment that is rapidly time-varying, as is the case in this study (Fig. 1A, where each 7 day labeling ramp and the subsequent washout are steep in both directions). In this type of situation, MIDA is an asset because it reveals the “effective” precursor enrichment, the integrated average of the precursor enrichments at which the individual palmitate molecules in a population were “born.” In principle, this is more appropriate than body water enrichments (Fig. 1A) for calculating fractional replacement (10). MIDA-based inference of the precursor enrichment is not, however, always necessary. The need for MIDA-based integrated or effective precursor enrichments can be obviated by water dosing strategies that more closely approximate a steady-state plateau, so that one can reasonably assume that all palmitate molecules were synthesized at a similar precursor label enrichment, which was not the case in the present study. Alternatively, an integrated mean precursor pool enrichment based on serial body water measurements can be used as a surrogate. This latter approach requires a priori knowledge of the general half-life of the product molecule to select a time period over which measured body water enrichments should be averaged.

### DNL is elevated in patients with NASH with cirrhosis

Data concerning DNL in cirrhotic patients with NASH have been limited. Rates of ^3^H-acetate and ^14^C-palmitate incorporation into triglycerides by liver from patients with progression to cirrhosis were lower when compared with patients with NAFLD/NASH but higher than in healthy individuals (28). Data from the current study demonstrate that DNL remains high in cirrhotic patients despite the reduction in liver fat content observed in this group. This finding suggests, first, that the remaining liver mass is still synthesizing new fatty acids in cirrhosis and, second, that treatment approaches aimed at suppressing lipogenesis may provide benefits in patients with NASH with cirrhosis.

### Interpatient variability and correlations with fibrotic and metabolic markers

Our data also demonstrate that DNL is positively correlated with circulating markers related to plasma TG and VLDL and with the lipoprotein insulin resistance score in both cirrhotic and noncirrhotic patients with NASH. These findings are consistent with recent results (27) in obese patients showing strong inverse correlations between hepatic DNL and whole-body as well as hepatic insulin sensitivity, measured by glucose clamp protocols, and strong direct correlations between hepatic DNL and 24 h plasma glucose and insulin values. The finding here of an inverse correlation with plasma beta-hydroxybutyrate levels is interesting and consistent with the notion that ketosis is inversely correlated with DNL, due to the opposing actions of malonyl-CoA on these two processes in the liver (29).

On the other hand, our data show that DNL is minimally correlated with circulating or imaging-based fibrosis markers in patients with noncirrhotic NASH. In our small cirrhotic cohort (n=20), there are moderate negative correlations observed between DNL and ELF. More DNL data from cirrhotic NASH populations will be needed to confirm this observation. It should be noted that our method measures DNL made in hepatocytes, as these are the source of lipids secreted from the liver into plasma and would not detect any DNL that occurs in stellate cells, which might influence fibrogenesis (30).

### Effects of DNL-targeted treatment in NASH can be determined in test-retest studies by using an individualized correction model

Measuring the effects of interventions on hepatic DNL is of obvious importance, but because hepatic TG turns over with a long half-life, labeling protocols of 1–2 weeks or more with heavy water are needed. An additional consequence is that TG in liver that was labeled by the DNL pathway prior to a repeat heavy water administration (e.g., the measured plasma palmitate enrichment at week 2, Fig. 1C) may contaminate plasma palmitate present after the repeat labeling protocol, whether or not the precursor pool (body water) is still enriched with label at the time of starting the second labeling protocol. This will interfere with accurate assessment of DNL with any labeled substrate, not only heavy water, during the treatment period, thereby potentially confounding the goal of repeat labeling studies.

Some correction clearly needs to be made, but one cannot simply subtract the baseline DNL value at the beginning of the repeat 2 week labeling protocol because some of this preexisting DNL in liver TG will have subsequently died away during the 2 weeks of repeat labeling (discussed in Methods). The need to measure what “would have been” present from prior DNL at the end of the repeat labeling period (if there had not been a repeat labeling protocol) presents a difficult dilemma.

We provide here a practical solution to this measurement problem, based on the two-pool model of liver TG kinetics. By measuring the label die-away rate in plasma palmitate after deuterium is substantially cleared from the body (e.g., 8 weeks after a labeling protocol), we were able to generate an individualized correction factor for each patient that allowed us to model and subtract the DNL value that would have been present if there had not been a repeat labeling protocol.

Our finding that the individually corrected treatment week 4 DNL values during treatment with the ACC inhibitor firsocostat are nearly identical to the treatment week 12 DNL values (which did not need to be corrected, as body water and plasma TG-palmitate enrichments had already returned to negligible levels) represents a strong validation of both the correction approach and the two-pool model. The implication is that early intervention effects, such as after 4 weeks of treatment, are compatible with use of labeling for test-retest studies, including but not limited to heavy water labeling, by use of this correction model.

In the current study, clinical sites were rigorously trained and DNL data were generated in the setting of a clinical trial from a large NASH study cohort (n=123) that contained both noncirrhotic and cirrhotic patients. The extensive clinical and biomarker data from the current study also enabled us to evaluate correlations between DNL and a variety of markers in study patients. Limitations from the current study include the relatively small NASH cirrhotic patient cohort (n=20) and the combination of invasive (biopsy) and noninvasive eligibility criteria, so there is some baseline variability in fibrosis stage. Subjects enrolled in this study were not advised to follow specific dietary instructions and food intake data were not collected, so we cannot assess the potential impact of diet on DNL. We have recently reported (31) that removal of free sugar from the diet of adolescent males with fatty liver disease significantly reduced both liver fat and DNL measured by a similar protocol as was used here. Comparisons here within subjects might mitigate potential effects of diet. The use of the individualized correction factor for the 4 week treatment time point also assumes that liver TG half-life between days 56 and 70 is not different from day 15–28 of the treatment period and is not altered by the treatment regimen.

The current findings may inform future studies in several ways. The high DNL contribution measured with longer labeling studies in patients with NASH represents further rationale for targeting DNL in these patients, in addition to providing guidance on how to accurately measure the contribution from DNL. The slow turnover time of liver TG pools in NASH also provides a sense of the general time frame over which any intervention can be expected to reduce liver fat: it is unlikely that this will be faster than the half-life of the hepatic storage pool, even if inhibition of TG deposition is immediate and highly effective. The use of an individualized correction factor allows effects of interventions to be assessed even when there is residual label in liver TG. Finally, the relations with liver fibrosis, high DNL persisting even in cirrhosis, despite lower total liver fat stores, and to markers of fibrosis may provide insight into the interaction between lipogenesis and fibrosis in this multifaceted disorder.

## Data availability

Gilead shares anonymized Individual Patient Data (IPD) upon request or as required by law and/or regulation with qualified external researchers. Approval of such requests is at Gilead’s discretion and is dependent on the nature of the request, the merit of the research proposed, the availability of the data, and the intended use of the data. Data requests should be sent to datarequest@gilead.com.

## Supplemental data

This article contains supplemental data (10, 31).

## Conflicts of interest

E. J. L., K. W. L., E. N., T. J. F. have no conflicts of interest; M. K. H. has received grant support and has consulted for Gilead; J.-C. C., A. B., L. W., Y. W., R. S. H., C. C., G. M. S., and R. P. M. are employees of Gilead and own Gilead stocks.

## Supplemental data

DNL in NASH patients JLR Reviewer Revised (Supplement)
