# Supplementary material for: Elevated de novo lipogenesis, slow liver triglyceride turnover, and clinical correlations in nonalcoholic steatohepatitis patients
Source: J Lipid Res. 2022 Jul 11;63(9):100250. doi: 10.1016/j.jlr.2022.100250 (PMC9424583; doi:10.1016/j.jlr.2022.100250)

**Supplementary Appendix to:**

**Elevated de novo lipogenesis, slow liver triglyceride turnover and clinical correlations in nonalcoholic steatohepatitis patients**

Kelvin W. Li, Eric J. Lawitz, Edna Nyangau, Tyler John Field, Jen-Chieh Chuang, Andrew Billin, Lulu Wang, Ya Wang, Ryan S. Huss, Chuhan Chung, G. Mani Subramanian, Robert P. Myers, and Marc K. Hellerstein

**Inclusion Criteria**

Subjects must meet all of the following inclusion criteria to be eligible for participation in this study.

1) Males and females between 18-75 years of age; inclusive based on the date of the Screening visit;

2) Willing and able to give informed consent prior to any study specific procedures being performed;

3) Meets all of the following conditions:

a) A clinical diagnosis of nonalcoholic fatty liver disease (NAFLD) with imaging documenting fatty liver within two years prior to Screening. If necessary an ultrasound may be performed during Screening to confirm NAFLD. Note: criterion 3a. must be met before evaluating criteria 3b. and 3c.

b) Screening MRI-PDFF with ≥10% steatosis

c) Screening MRE with liver stiffness ≥2.90 kPa

4) Platelet count ≥100,000/mm^3^

5) Creatinine Clearance (CLcr ) as calculated by the Cockcroft-Gault equation ≥60 ml/min

6) Female subjects of childbearing potential must have a negative serum pregnancy test prior to starting study treatment;

7) All female subjects of childbearing potential who engage in heterosexual intercourse must agree to use a highly effective method of contraception during intercourse from the Screening visit throughout the study period and for 30 days following the last dose of study drugs;

8) Male subjects are required to use barrier contraception (condom plus spermicide) during intercourse from the Screening through the study completion and for 90 days following the last dose of study drugs;

9) Male subjects must refrain from sperm donation from Screening through at least 90 days following the last dose of study drugs.

10) Female subjects must refrain from egg donation or harvest for 30 days after last dose of study drugs.

11) Willing and able to comply with scheduled visits, drug administration plan, laboratory tests, other study procedures, and study restrictions.

12) Must be able to read and complete Quality of Life questionnaires independently.

**Exclusion Criteria**

Subjects who meet any of the following exclusion criteria will not be randomized in this study.

1) Pregnant or lactating females; lactating females must agree to discontinue nursing before the study drugs are administered;

2) ALT >5 × ULN;

3) Other causes of liver disease including autoimmune, viral, and alcoholic liver disease;

4) Cirrhosis of the liver as defined by any of the following:

a) Cirrhosis on historical liver biopsy (e.g. Brunt/Kleiner stage 4 or equivalent);

b) Evidence of cirrhosis on liver imaging (e.g. ultrasound, CT, or MRI) including a nodular liver surface, splenomegaly, or portal venous collaterals;

c) Screening FibroSURE/FibroTest ≥0.75, as determined by the central laboratory;

d) Prior history of decompensated liver disease, including ascites, hepatic encephalopathy or variceal bleeding;

5) History of liver transplantation;

6) Weight reduction surgery in the past or planned during the study (weight reduction surgery is disallowed during the study);

7) History of intestinal resection or malabsorptive condition that may limit the absorption of GS-0976. Prior cholecystectomy and appendectomy are permitted

8) BMI <18 kg/m^2^;

9) INR >1.2 unless on anticoagulant therapy;

10) Total bilirubin >1 × ULN, except with diagnosis of Gilbert’s syndrome;

11) Chronic hepatitis B (HBsAg positive);

12) Chronic hepatitis C (HCV Ab and HCV RNA positive);

13) HIV Ab positive;

14) Alcohol consumption greater than 21 units/week for males or 14 units/week for females. (A unit is 10 ml of pure alcohol. 1-1.5 units of alcohol are present in 0.5 pint of beer, 1 4-oz/120-mL glass of wine, and a standard pub measure of 40% proof alcohol);

15) Positive urine screen for amphetamines, cocaine or opiates (i.e. heroin, morphine) at Screening. Subjects on stable methadone or buprenorphine maintenance treatment for at least 6 months prior to Screening may be included in the study. Subjects with a positive urine drug screen due to prescription opioid-based medication are eligible if the prescription and diagnosis are reviewed and approved by the investigator;

16) Unstable cardiovascular disease as defined by any of the following:

a) Unstable angina within 6 months prior to Screening

b) Myocardial infarction, coronary artery bypass graft surgery or coronary angioplasty within 6 months prior to Screening

c) Transient ischemic attack or cerebrovascular accident within 6 months prior to Screening

d) Obstructive valvular heart disease or hypertrophic cardiomyopathy

e) Congestive heart failure;

17) Use of prohibited concomitant medications as described in Section 5.4

18) History of a malignancy within 5 years prior to Screening with the following exceptions:

a) Adequately treated carcinoma in situ of the cervix

b) Adequately treated basal or squamous cell cancer or other localized non-melanoma skin cancer;

19) Any laboratory abnormality or condition that, in the investigator’s opinion, could adversely affect the safety of the subject or impair the assessment of study results;

20) Participation in another investigational study of a drug or device within 1 month prior or within 5 half-lives of the prior investigational agent (whichever is longer) prior to Screening;

21) Concurrent participation in another therapeutic clinical study;

22) Known hypersensitivity to GS-0976, the metabolites, or formulation excipient;

23) Presence of any condition that could, in the opinion of the investigator, compromise the subject’s ability to participate in the study, such as history of substance abuse or a psychiatric (including any subjects with a psychiatric hospital admission or emergency room visit in the 2 years prior to Screening) or medical condition;

24) Unavailable for follow-up assessment or concern for subject’s compliance with the protocol procedures;

25) Contraindications or inability to complete MRI scanning (e.g. presence of permanent pacemakers, implanted cardiac devices, weight restrictions, etc.).

**Supplementary Figure 1**. Study design.

**Supplementary table 1**. Associations of DNL with other markers in NASH patients

| Demographics | | | | | | |
| --- | --- | --- | --- | --- | --- | --- |
|  | **All patients**  **(n=123)** | | **Patients without Cirrhosis (n=103)** | | **Patients with Cirrhosis (n=20)** | |
|  | Correlation  (r) | p value | Correlation  (r) | p value | Correlation  (r) | p value |
| Age | -0.09 | 0.33 | -0.07 | 0.509 | -0.02 | 0.94 |
| Baseline BMI | 0.1 | 0.27 | 0.18 | 0.073 | -0.58 | 0.01 |
| Liver Biochemistry | | | | | | |
| Alanine Aminotransferase (U/L) | -0.06 | 0.53 | -0.16 | 0.115 | 0.41 | 0.07 |
| Aspartate Aminotransferase (U/L) | -0.07 | 0.41 | -0.1 | 0.335 | 0.17 | 0.48 |
| Gamma Glutamyl Transferase (U/L) | -0.01 | 0.87 | -0.05 | 0.583 | 0.41 | 0.07 |
| Alkaline Phosphatase (U/L) | -0.14 | 0.12 | -0.14 | 0.164 | -0.09 | 0.72 |
| Albumin (g/dL) | -0.14 | 0.14 | -0.25 | 0.011 | 0.5 | 0.02 |
| Metabolic factors | | | | | | |
| Fasting Glucose (mg/dL) | 0.16 | 0.09 | 0.06 | 0.53 | 0.63 | 0.00 |
| Fasting Homostat Model Assess of Insulin Rstn | 0.18 | 0.05 | 0.15 | 0.12 | 0.41 | 0.07 |
| Hemoglobin A1C (%) | 0.04 | 0.69 | -0.06 | 0.52 | 0.48 | 0.03 |
| Fasting Insulin (uIU/mL) | 0.14 | 0.13 | 0.16 | 0.12 | 0.15 | 0.54 |
| Proinsulin (pmol/L) | 0.2 | 0.02 | 0.16 | 0.10 | 0.55 | 0.01 |
| Platelets (x10^3/uL) | 0.18 | 0.05 | 0.14 | 0.17 | 0.29 | 0.22 |
| Bilirubin (mg/dL) | -0.13 | 0.16 | -0.11 | 0.25 | -0.17 | 0.48 |
| Direct Bilirubin (mg/dL) | -0.13 | 0.15 | -0.09 | 0.35 | -0.27 | 0.25 |
| Fasting Bile Acid (umol/L) | -0.18 | 0.06 | -0.14 | 0.16 | -0.31 | 0.18 |
| Fibroblast Growth Factor 19 (pg/mL) | -0.03 | 0.77 | 0.02 | 0.87 | -0.2 | 0.39 |
| 7-Alpha-Hydroxy-4-Cholesten-3-One (ng/mL) | 0.06 | 0.50 | -0.03 | 0.74 | 0.42 | 0.06 |
| Plasma Lactate Dehydrogenase (mg/dL) | 0.27 | 0.00 | 0.25 | 0.01 | 0.59 | 0.01 |
| Fasting Triglycerides (mg/dL) | 0.28 | 0.00 | 0.24 | 0.02 | 0.49 | 0.03 |
| HDL Cholesterol (mg/dL) | -0.13 | 0.17 | -0.11 | 0.26 | -0.27 | 0.26 |
| Non-HDL Cholesterol (mg/dL) | 0.16 | 0.07 | 0.12 | 0.23 | 0.28 | 0.23 |
| VLDL Triglycerides (mg/dL) | 0.29 | 0.00 | 0.23 | 0.02 | 0.58 | 0.01 |
| Total HDL Particles (umol/L) | 0.11 | 0.24 | 0.05 | 0.62 | 0.31 | 0.18 |
| Total LDL Particles (nmol/L) | 0.1 | 0.26 | 0.04 | 0.66 | 0.3 | 0.21 |
| Total VLDL & Chylomicron Particles (nmol/L) | 0.15 | 0.10 | 0.08 | 0.43 | 0.55 | 0.01 |
| Apolipoprotein A1 (mg/dL) | -0.07 | 0.47 | -0.07 | 0.45 | -0.17 | 0.47 |
| Apolipoprotein B (mg/dL) | 0.15 | 0.11 | 0.09 | 0.39 | 0.33 | 0.15 |
| Lipoprotein Insulin Resistance Score | 0.37 | 0.00 | 0.37 | <0.001 | 0.37 | 0.11 |
| Large VLDL (nmol/L) | 0.42 | 0.00 | 0.41 | <0.001 | 0.5 | 0.02 |
| Medium VLDL (nmol/L) | 0.13 | 0.14 | 0.07 | 0.48 | 0.5 | 0.03 |
| Small VLDL (nmol/L) | 0.02 | 0.82 | -0.05 | 0.64 | 0.51 | 0.02 |
| HDL Size (nm) | -0.17 | 0.06 | -0.13 | 0.19 | -0.33 | 0.16 |
| LDL Size (nm) | -0.05 | 0.56 | -0.04 | 0.71 | -0.17 | 0.47 |
| VLDL Size (nm) | 0.41 | 0.00 | 0.44 | <0.001 | 0.22 | 0.36 |
| Adiponectin (ng/mL) | -0.15 | 0.10 | -0.09 | 0.39 | -0.57 | 0.01 |
| Leptin (pg/mL) | -0.04 | 0.63 | -0.01 | 0.93 | -0.37 | 0.11 |
| Fasting Free Fatty Acid (mEq/L) | -0.14 | 0.13 | -0.19 | 0.06 | 0.08 | 0.73 |
| Beta-Hydroxybutyrate (mg/dL) | -0.27 | 0.00 | -0.32 | 0.00 | 0.1 | 0.68 |
| Imaging | | | | | | |
| MRI-PDFF (%) | 0.03 | 0.78 | -0.02 | 0.81 | 0.25 | 0.29 |
| Magnetic Resonance Elastography (kPa) | -0.07 | 0.46 | 0.01 | 0.94 | -0.19 | 0.43 |
| Serum Markers of fibrosis and inflammation | | | | | | |
| Fibrosis-4 Score | -0.18 | 0.04 | -0.15 | 0.13 | -0.32 | 0.17 |
| Fibrosure/Fibrotest Score | -0.16 | 0.07 | -0.12 | 0.24 | -0.26 | 0.28 |
| AST to Platelet Ratio Index | -0.16 | 0.07 | -0.16 | 0.10 | -0.11 | 0.64 |
| Enhanced Liver Fibrosis Score | -0.09 | 0.33 | 0 | 0.98 | -0.52 | 0.02 |
| Hyaluronic Acid (HA; ng/mL) | -0.08 | 0.35 | 0.01 | 0.89 | -0.54 | 0.02 |
| Procollagen III N-Terminal Propeptide (PIIINP; ng/mL) | -0.13 | 0.15 | -0.08 | 0.45 | -0.31 | 0.18 |
| Tissue Inhibitor of Metalloproteinase 1(TIMP1; ng/mL) | 0.06 | 0.51 | 0.11 | 0.28 | -0.04 | 0.86 |
| Keratin, Type I Cytoskeletal 18, M30 (U/L) | 0.08 | 0.41 | 0.07 | 0.50 | 0.03 | 0.89 |
| Keratin, Type I Cytoskeletal 18, M65 (U/L) | 0.06 | 0.55 | 0.04 | 0.67 | 0.18 | 0.44 |
| Fasting C-Peptide (ng/mL) | 0.13 | 0.15 | 0.16 | 0.12 | 0.15 | 0.53 |
| Plasminogen Activator Inhibitor 1 (ng/mL) | 0.18 | 0.05 | 0.17 | 0.10 | 0.24 | 0.32 |
| Tumor Necrosis Factor (pg/mL) | -0.03 | 0.70 | -0.03 | 0.78 | 0.05 | 0.83 |
| Interleukin-6 (pg/mL) | 0.1 | 0.26 | 0.18 | 0.07 | -0.23 | 0.32 |
| C-C Motif Chemokine 2 (pg/mL) | 0.08 | 0.38 | 0.13 | 0.19 | -0.23 | 0.32 |
| Transforming Growth Factor Beta-1 (ng/mL) | 0.17 | 0.07 | 0.14 | 0.17 | 0.31 | 0.18 |


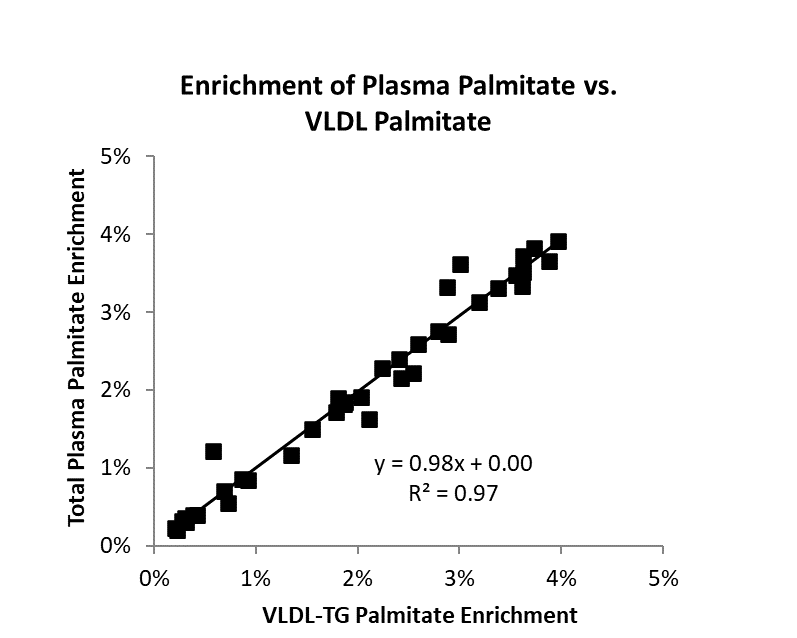


**Supplemental Figure 2.** For a subset of fasting plasma samples collected in this study, VLDL particles were isolated from plasma by sequential ultracentrifugation, total lipids were extracted from VLDL with chloroform:methanol (2:1), and VLDL-triacylglycerols (VLDL-TGs) were then isolated by thin layer chromatography, as described previously (31). VLDL-TG fatty acids were transesterified to fatty acid-methyl esters for gas chromatography-mass spectrometry analyses, and isotopic enrichments (%EM1) were compared to those of total plasma palmitate, collected as described in the Methods.

**Supplemental Figure 3.** Time Course of De novo lipogenesis (DNL) over 14 days of heavy water labeling in NASH patients with cirrhosis given Firsocostat for 12 weeks, with repeat measurements of DNL at weeks 4 and 12 of treatment. A 2-phase exponential curve fit is shown as the connecting line. Data are mean±SD (n=10).

**Sample Calculations for %DNL**

*Equations for MIDA p (as a function of EM2/EM1) and %EM1* (as a function of MIDA p).*

Versions of these equations have been presented in previous publications (10).

MIDA simulation of the enrichment of the 270 ion of methyl palmitate (C17H34O2), assuming 21 potential sites for covalent deuterium labeling by D2O (n), was performed for a range of deuterium enrichments in the body (p). This simulation provided the data for curve fit equations that allowed us to interpolate the measured EM2/EM1 ratios into the p that must have generated them.


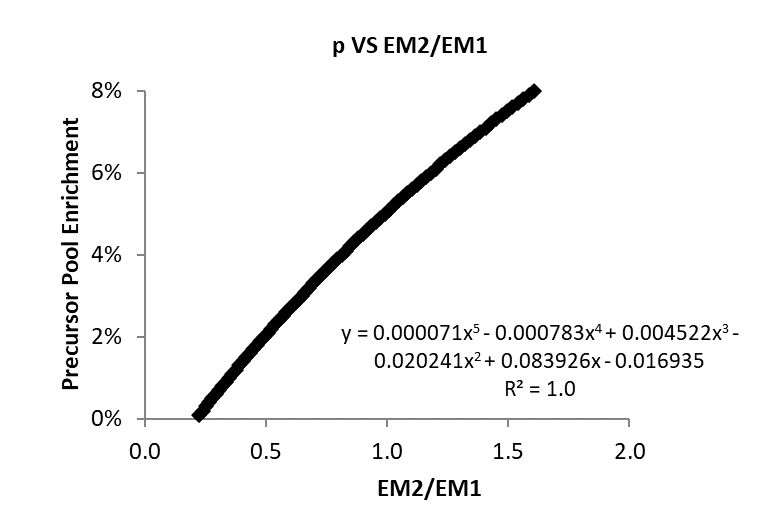


From the p values that were computed from EM2/EM1 values in labeled samples of methyl palmitate by MIDA (precursor pool enrichment), the asymptotic EM1* were calculated for use in the equations to calculate DNL.


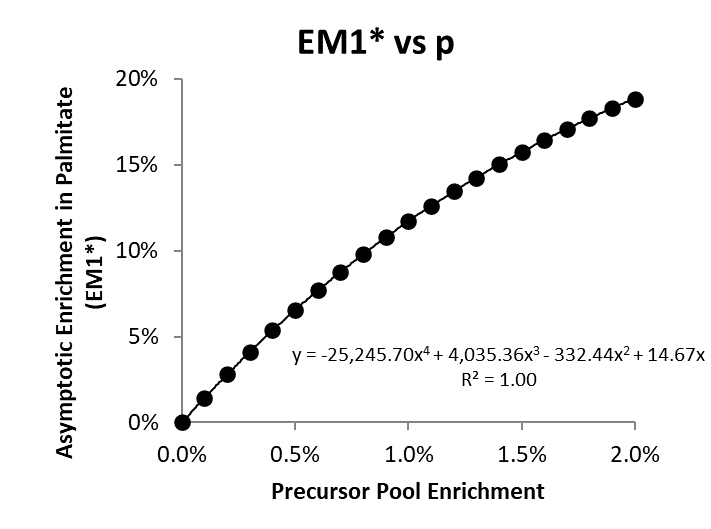

Supplement: DNL in NASH patients JLR Reviewer Revised (Supplement) [file mmc1.docx]
